# Learning unsupervised contextual representations for medical synonym discovery

**DOI:** 10.1093/jamiaopen/ooz057

**Published:** 2019-11-04

**Authors:** Elliot Schumacher, Mark Dredze

**Affiliations:** Department of Computer Science, Johns Hopkins University, Baltimore, Maryland, USA

**Keywords:** synonym discovery, contextual representations, medical terminology

## Abstract

**Objectives:**

An important component of processing medical texts is the identification of synonymous words or phrases. Synonyms can inform learned representations of patients or improve linking mentioned concepts to medical ontologies. However, medical synonyms can be lexically similar (“dilated RA” and “dilated RV”) or dissimilar (“cerebrovascular accident” and “stroke”); contextual information can determine if 2 strings are synonymous. Medical professionals utilize extensive variation of medical terminology, often not evidenced in structured medical resources. Therefore, the ability to discover synonyms, especially without reliance on training data, is an important component in processing training notes. The ability to discover synonyms from models trained on large amounts of unannotated data removes the need to rely on annotated pairs of similar words. Models relying solely on non-annotated data can be trained on a wider variety of texts without the cost of annotation, and thus may capture a broader variety of language.

**Materials and Methods:**

Recent contextualized deep learning representation models, such as ELMo (Peters et al., 2019) and BERT, (Devlin et al. 2019) have shown strong improvements over previous approaches in a broad variety of tasks. We leverage these contextualized deep learning models to build representations of synonyms, which integrate the context of surrounding sentence and use character-level models to alleviate out-of-vocabulary issues. Using these models, we perform unsupervised discovery of likely synonym matches, which reduces the reliance on expensive training data.

**Results:**

We use the ShARe/CLEF eHealth Evaluation Lab 2013 Task 1b data to evaluate our synonym discovery method. Comparing our proposed contextualized deep learning representations to previous non-neural representations, we find that the contextualized representations show consistent improvement over non-contextualized models in all metrics.

**Conclusions:**

Our results show that contextualized models produce effective representations for synonym discovery. We expect that the use of these representations in other tasks would produce similar gains in performance.

## INTRODUCTION

Given a word or phrase, synonym discovery identifies other words or phrases that have the same or similar meaning to the original. Constructing lists of synonyms can be helpful in a range of downstream applications, such as linking concepts to a knowledge base^3^ or query expansion in information retrieval. Both tasks rely on an expanded list of synonyms to ensure that relevant concepts or documents are retrieved even if they do not contain the query term. Synonym discovery, and the related task of paraphrase identification,^4–6^ have been explored using a variety of methods.^7–10^ This task builds on work in measuring semantic similarity between words and phrases.^11^^,^^12^

Synonym discovery is especially important within the clinical medical domain.^13–15^ Medical synonyms aid in a variety of clinical tasks, such as medical concept linking,^16^ automatic phenotyping, and cohort selection for comparative effectiveness research.^17^ However, while ontologies contain synonyms for a specific term, these often do not contain the variety of synonyms that can occur in clinical notes across different authors and different domains. Alternatively, new or rare terms may not be present in a standardized ontology. As these types of terms are by definition not present in annotated data, models which can leverage large unannotated datasets to infer meaning from surrounding text are critical in representing these terms. An additional challenge is that a fixed synonym list may not accurately reflect meaning, as abbreviations and shortened references have meanings that are contextually dependent. For these reasons, we are interested in methods that can automatically identify new synonyms from clinical notes without supervision.

In this work, we consider the task of identifying whether 2 textual mentions of a disorder refer to the same underlying medical concept. While synonym discovery is critical within clinical NLP, the task is especially challenging for disorder mentions in the clinical domain. First, while typical synonyms tend to be lexically dissimilar, medical concepts are both lexically similar (“dilated RA” and “dilated RV”) or dissimilar (“cerebrovascular accident” and “stroke”). A solution to this problem is to use representations of terms that move away from using the lexical items themselves. Along these lines, work by Wang et al^15^ proposed word embeddings for this task, and found that Word2vec representations improved over the previous best approach. However, type level representations cannot address the second challenge: synonym determination is often contextual. For example, the 2 terms “diabetes type 2” and “diabetes” can be synonymous in that they refer to the same underlying concept, or they could refer to 2 different types of diabetes. Within an ontology, there exists other relationships (eg hypernym, hyponym) beyond synonymy. However, we focus solely on synonyms as this allows for a more precise evaluation. The key distinguishing factor is the context of how the terms appear in the clinical note. Context can come from both the surrounding text, as well as information about the patient. Without this context, a method cannot distinguish when 2 disorder mentions are synonymous.

We propose learning representations of clinical text for unsupervised synonym discovery of disorder mentions using contextualized representations. Rather than build type level embeddings as in previous work,^15^ we build on recent work in learning contextualized text representations.^1^^,^^2^^,^^18^ These methods incorporate the mention context into a representation of the mention. Additionally, we augment the context from learned representations of the patient.^19^ Incorporating context from the patient record can indicate that certain concepts are more or less likely for a mention. Additionally, our methods are fully unsupervised, in contrast to the previous work that used supervision.^15^ We greatly prefer unsupervised methods as they can scale to a large number of medical subdomains as medical annotations are particularly expensive.

We evaluate our proposed method on the task of finding disorder synonyms^20^ from English clinical free text, where we define mentions as synonymous if they both refer to the same medical concept. We consider baselines of both unlearned representations (character ngrams), and learned non-contextualized word embeddings (Word2vec). We show improvements on the dataset released for ShARe/CLEF eHealth Evaluation Lab 2013 Task 1b,^21^ which includes span-level annotations for disorder concepts built on a subset of MIMIC 2.5 clinical notes. We find that both text context and patient context improve over previously established baselines, yielding significant improvements in the state of the art. Finally, we find that our methods identify synonyms that are more lexically dissimilar than Word2vec.

## SYNONYM DISCOVERY

We consider the task of finding disorder synonyms^20^ in English clinical free text. We define mentions as synonymous if they both refer to the same medical concept. To obtain annotations of this task, we identify mentions that link to the same medical concept in the dataset released for ShARe/CLEF eHealth Evaluation Lab 2013 Task 1b,^21^ which includes span-level annotations for disorder concepts built on a subset of MIMIC 2.5 clinical notes. For training representations, we use MIMIC III^22^ which is a superset of MIMIC 2.5.

Consider the sentence “The patient showed signs of a *stroke*” and the sentence “The patient’s father previously had a *cerebrovascular accident* at the same age.” The annotated dataset links both mentions (italicized) to the same ontology concept Cerebrovascular accident. From this we derive that these mentions are synonymous.

The contextual information of the mention is vital in correctly identifying the right synonym. For example, the mention stroke could also refer to the concept *Heat Stroke*, and further information is needed to identify the correct synonym. The surrounding text of the mention is one key source of information. Considering the previous example, the presence of the term hemorrhage would likely indicate the mention is synonymous with Cerebrovascular accident and not Heat Stroke. Additionally, patient information is often a relevant indicator. If the patient is an infant, Heat Stroke may be more likely than Cerebrovascular accident due to the higher likelihood of Heat Stroke in that population. Our goal is to learn representations that capture the synonymous relationship between the mention and concept by incorporating the context.

We formalize synonym discovery as follows. For a dataset of *N* medical records, each record *d* corresponds to patient *p* and contains zero or more highlighted textual mentions *m* linked to medical concept *c*. We then learn a representation of each *m* in the corpus. To construct the candidate synonym list, we consider each mention as a query *m_q_* and rank all other mentions as candidate synonyms *m_c_* based on cosine similarity, and consider the top *k *=* *50 mentions to be candidate synonyms to *m_q_*. We measure the effectiveness of our approach by ranked list quality, where a correct synonym is one where *m_q_* and *m_c_* share medical concept *c*.

Previous work^15^ investigated creating type level representations for synonym discovery using Word2vec continuous bag of words model (CBOW).^23^ They utilized a semi-supervised variant of this method for synonym discovery, and found it to be the best performing model they considered. However, this method does not consider context and does not yield representations specific to individual tokens. We consider several methods that capture the context in which the word occurs, and that may be more likely to identify synonyms with divergent lexical forms. Additionally, we integrate learned representations of patient data, specifically diagnosis codes, which can provide additional context. To focus on synonym discovery we assume gold mention spans.

### Bidirectional neural language models

Work by Peters et al^1^ proposed a different model for building representations of words that integrates the surrounding sentence context. ELMo (Embeddings from Language Models) utilizes recurrent neural networks to learn word representations in context by training them using language model objectives. These models were validated by achieving state of the art performance on a range of NLP tasks.

ELMo representations are trained using a neural Bidirectional Language Model (BiLM), which models the forward language model probability of a token *t_k_* given its history (*t_1_*, …, *t_k-1_*). The model computes a context-independent token representation $x_{k}^{\mathrm{LM}}$, using a convolutional neural network over the token’s characters. The token representation is passed through *L = 2* layers of an LSTM—the final layer is used to predict the next token using a softmax layer. The backward language model is the same, except the probability of token t_k_ is trained given its future context (*t_k+1_*, … *t_N_*), and the final layer predicts the previous token. The parameters for the token representation and the softmax layer are tied between the forward and backward models, while all other LSTM parameters are independent.

After training the BiLM model, representations for each word in a sentence are built by passing an entire sentence through the language model, and recording the resulting layers at each time step. This results in sentence-specific representations for each word, as opposed to the general representations in Word2vec or Context2vec. For each word, there are 3 representations—the token representation (referred to as layer 0), the intermediate representation from the first layer of the LSTM (layer 1), and the final representation resulting from the top layer of the LSTM (layer 2). Both representations from the LSTM, layers 1 and 2, are the result of concatenating the respective representations from the forward and backward LSTMs. Each of these representations provides different types of information about the word. The authors note that the second layer is most effective for word sense disambiguation, a semantically-oriented task, whereas the token representation is more lexically-oriented.

The representations for the ELMo model have several different characteristics—first, it uses a character model which enables it to build representations of out of vocabulary words. Additionally, to create a representation for a mention, it processes the entire sentence containing the mention through BiLM, not only the mention text. This allows for the representations for the words in the sentence to be informed by the immediate surrounding context. Therefore, the representation for a given mention may be different in 2 distinct sentences, even if the words are the same. For mentions containing multiple words, we explore using both the dimensional average and maximum to create a single mention representation. As ELMo uses a character model, out-of-vocabulary words are not an issue.

### Bidirectional transformer models

Later work in neural language models include BERT (Bidirectional Encoder Representations from Transformers).^2^ Unlike ELMo, which represents the right and left context by 2 separate LSTMs, BERT jointly conditions on both. Instead of character embeddings, BERT takes Token, Segment, and Positional embeddings as input. These are fed through several layers of bidirectional transformers.

### Patient medical context

Context2vec, ELMo, and BERT all utilize context from the text in some form. However, clinical medical records contain extensive structured data. ICD-9 (International Classification of Diseases) diagnostic codes are commonly used to represent a patient since they indicate symptoms and diagnosed conditions. Our corpus is a large de-identified medical records dataset. It contains one or more text records for a single hospital admission for a patient, and a set of ICD-9 codes that applies to the admission overall (As noted on MIMIC’s website, all ICD codes in MIMIC III are in ICD-9 format, and these codes will be switched to ICD-10 in later releases. Our model is not specific to ICD-9 code format—a representation for ICD-10 or ICD-11 codes could be learned in a similar way.). We utilize the Med2vec^19^ toolkit in order to learn representations of patient codes. For each admission, the assigned codes are converted into a binary vector representing all present codes. The binary vector representation is fed into a second hidden layer, which is concatenated with a vector containing demographic information about the patient. This in turn is fed into a final output layer, which is trained to predict neighboring visits using a skip-gram architecture. We use the provided author’s code to train it on patient data taken MIMIC III.

**Figure 1.**
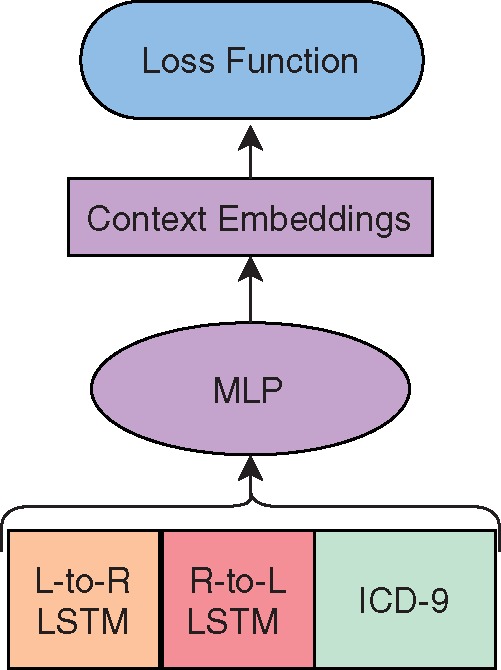
Architecture for Context2vec with ICD-9 representation integration.

We integrate Med2vec as patient-level context into both the Context2vec and ELMo models, in the hope that providing additional patient-level context will improve the representations of the words. We omit integration with BERT as it performs worse than ELMo in our tuning set (see Table 1). We integrate Med2vec with Context2vec by concatenating a single vector of averaged ICD-9 code embeddings for the linked hospital admission (noted as v) to the final states of both LSTMs, passing the combined vector as input into the final multi-layer perceptron (MLP) in the model. The input to the MLP becomes

$$\mathrm{lLS}(l_{1:i-1})⊕\mathrm{rLS}(r_{n:i+1})⊕\mathrm{ICD}9(v)$$

where lLS and rLS are left to right and right to left LSTM embeddings of the sentence up to the target word, respectively. By combining the ICD-9 context with the sentence context as input to a MLP, the model may learn patient-level context that informs the word-level representations created by Context2vec. The architecture is shown in Figure 1.

**Table 1.** Mean reciprocal rank, coverage, and top-1 accuracy, for pairwise identification of synonyms of the top 50 results run on the tuning data (*n* = 1275)

Model	MRR	Cov. (%)	Top-1 (%)
**Word2vec**	0.373	71.90	30.90
Char. Bigram	0.404	70.60	33.70
***Char. Trigram***	0.417	69.80	33.70
Char. Fourgram	0.414	68.80	34.00
Context2vec	0.374	58.10	31.80
***C2v + m2v***	0.385	63.90	28.40
ELMo			
L0, Avg	0.499	71.90	43.70
*** L0, Max***	0.503	67.80	**45.30**
L1, Avg	0.37	71.50	28.50
L1, Max	0.344	63.30	27.40
L2, Avg	0.299	71.10	21.90
L2, Max	0.291	63.70	22.20
ELMo + M2v In			
*** L0, Avg***	**0.504**	**76.20**	44.50
L0, Max	0.488	69.80	43.50
L1, Avg	0.346	72.20	26.50
L1, Max	0.336	65.50	26.90
L2, Avg	0.279	70.00	19.80
L2, Max	0.281	63.00	21.10
ELMo + M2v Out			
L0, Avg	0.484	72.20	42.60
*** L0, Max***	0.493	69.60	44.90
L1, Avg	0.371	73.30	29.10
L1, Max	0.351	66.80	27.70
L2, Avg	0.309	71.10	23.20
L2, Max	0.302	64.50	23.10
BERT			
*** L1, Avg***	0.491	65.18	43.92
L1, Max	0.489	65.02	44.31
L4, Avg	0.438	61.96	39.37
L4, Max	0.435	63.29	38.43
L8, Avg	0.340	53.96	30.90
L8 Max	0.395	51.69	35.53
L12, Avg	0.324	47.22	29.33
L12, Max	0.372	50.67	33.25

*Note*: For ELMo and BERT models, L(0/1/2) indicates layer number and Avg or Max indicates combination method for multiple word phrases. Bolded entries are the best performing result for that measure. We report test results on the model names listed in bolded italics, selecting the best model for MRR in each category (noted by line separators).

**Table 3.** Selected correct Top-1 examples from the ELMo (L0, Max) Model tuning set results

Ex. mention	Top-1 synonym
Varicosities	Varices
Left atrial enlargement	LA enlargement
Difficulty…breathing	Shortness of breath
Hypokinesis	Hypokinetic
Decreased responsiveness	Poorly responsive
Uterine fibroid	Fibroid
Mitral regurgitation	Mitral regurg
Rib fx	Fractures…rib
Septic	Sepsis

We explore 2 approaches to integrating Med2vec into ELMo: in the input layer and the output layer (We also experimented with multi-task training, but initial results did not show an improvement.). In both cases, we create a single ICD-9 representation by calculating the dimensional max over the Med2vec representations of each ICD-9 code, as there are multiple ICD-9 codes assigned to each note. First, we concatenate a matrix of ICD-9 codes to the token representation layer as input to the LSTM. Each position in the token representation layer $h_{k,\;0}^{\mathrm{LM}}$ becomes $h_{k,\;0}^{\mathrm{LM}}⊕\mathrm{ICD}9\left(v\right).$

Second, we add ICD-9 representations to the output layer as additional input to the softmax which predicts the preceding or future tokens. Previous work on neural language modeling^24^ has shown that integrating additional information into the language model can lower perplexity, specifically when added to the output layer. While our goal isn’t to improve language model perplexity, added information may similarly inform our task. Instead of the softmax input being the concatenated second layers from the forward and backward LSTMs, $h_{k,\;2}^{\mathrm{LM}}\;=h_{k,\;2}^{\mathrm{left}}⊕h_{k,\;2}^{\mathrm{right}},\;$we add the ICD-9 codes to yield

$$h_{k,\;2}^{\mathrm{LM}}\;=h_{k,\;2}^{\mathrm{left}}⊕h_{k,\;2}^{\mathrm{right}}\;⊕\mathrm{ICD}9(v)$$

**Figure 2.**
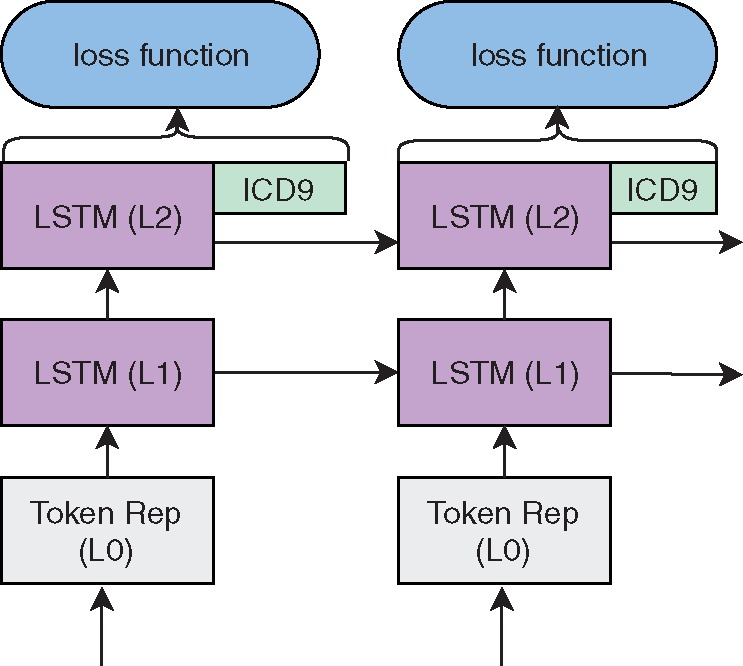
Architecture for ELMo with Med2vec representation integration in output layer.

**Figure 3.**
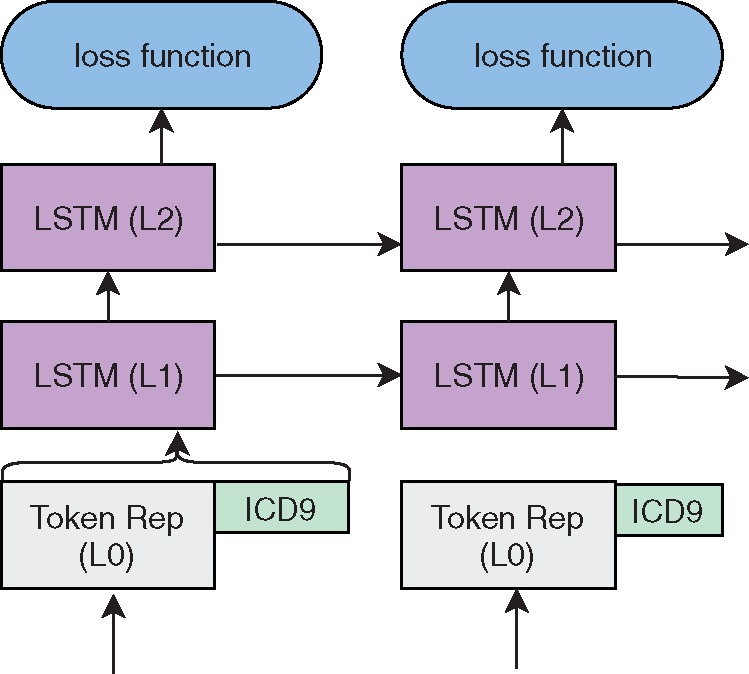
Architecture for ELMo with Med2vec representation integration in input layer.

The ICD-9 code matrix consists of a single ICD9 representation for each word (taken from the ICD9 codes linked to the clinical note). For the input version, the ICD9 representations are separately input into the respective LSTMs, while in the output only one matrix representation is included. In all cases, a single ICD-9 representation is created by calculating the dimensional max operation over the representations of the ICD-9 codes assigned to the admission. ELMo with Med2vec Output is illustrated in Figure 2 and ELMo with Med2vec Input is illustrated in Figure 3.

## DATA

We use 2 datasets: MIMIC III^22^ to train our representations, and the concept linking dataset released for ShARe/CLEF eHealth Evaluation Lab 2013 Task 1b^21^ to evaluate discovered synonyms. At the time of this research, this dataset is the only relevant English dataset publicly available, although a dataset used as part of the n2c2 2019 shared task will be released in the next year. The shared task dataset consists of span-level annotations for disorder concepts built on a subset of MIMIC 2.5 clinical notes^25^ (which is a subset of the current version, MIMIC III^22^) Since we evaluate an unsupervised method, we use a “tuning” set as it is only used to tune model hyperparameters, while the test set is for evaluating synonyms. The “training” dataset is only used for training the representations and consists of unannotated clinical notes. Two types of mention annotations are excluded—any concepts not included in the SNOMED-CT Disorder Semantic group or in the Finding, Body Substance, and Mental Process semantic types (We include all preferred entries, with the default settings of UMLS 2011AA, in the SNOMED-CT Disorder Semantic group (accounting for 116 436 unique concepts), but also include the first non-preferred entries that do not have a preferred entry (accounting for 8926 unique concepts).), and any concepts that have a non-concept annotation other than CUI-less. For our synonym task, for a mention *m_q_*, we consider another mention *m_c_* to be a synonym if they are linked to the same concept with different mention strings.

We train our representations on a subset of MIMIC III. As the annotated clinical notes are in the dataset, we excluded any patients that had an annotated clinical note from our representation tuning data. We used 213 466 clinical notes and associated admissions-level diagnoses data for training representations. For Med2vec, similarity is reported using the admission-level ICD-9 code representation, and does not use any of the clinical note text. We use an embedding dimensionality of 600 for Context2vec, and 200 for Med2vec, which was chosen based on the dimensionality used in the Context2vec and Med2vec papers, respectively. The Context2vec + Med2vec reported model was produced after 6 epochs of training, and we used the default setting for all other model parameters as noted in their respective papers. For ELMo, we used the standard parameters noted in the original paper—we trained for 10 epochs, with a dimensionality of 512 for each LSTM. For BERT, we used the pretrained model provided by,^26^ trained on a variety of clinical notes in MIMIC.

## EVALUATION

### Baselines

We include 2 baselines drawn from the work of Wang et al^15^ to serve as the state of the art methods for learned and unlearned representations. We do not evaluate their supervised model as we consider the unsupervised setting. Limited annotated data for synonyms exists, and we therefore believe that the unsupervised setting is more appropriate for this data.

#### Character ngrams

We calculate the number of *n*-length sequences of characters that appear in both the mention string and the candidate synonym. Each word is padded with unique start and end characters. Each mention-candidate pair is assigned a score equal to $\left(|{\mathrm{ngram}}_{c}\cap {\mathrm{ngram}}_{q}|)/(|{\mathrm{ngram}}_{q}|\right)$, where *ngram_q_* is the set of ngrams from the query mention and ngram_c_ is the set of ngrams from the candidate synonym. This score is used to create a ranking of synonyms, as with all other models.

#### Word2vec

Word2vec performed best in the unsupervised setting in previous work.^15^ We use Word2vec^23^ to learn representations of mention strings, using the MIMIC III corpus as training data. We used the gensim toolkit.^27^ We use negative sampling combined with the skipgram training algorithm, which are the best performing parameters from previous work.^15^ For mentions containing multiple words, the dimensional average of all words is used as a single mention representation. All out-of-vocabulary words are excluded from the final representation as Word2vec cannot provide representations for novel words, although this situation was rare.

### Evaluation metrics

For a single mention, we calculate the cosine similarity between that mention’s representation and all other mention representations, creating a ranking. From this, we take the top 50 mentions as potential synonym candidates and calculate mean reciprocal rank (MRR) and coverage. We exclude mention pairs that are equivalent strings as they are the same terms, though identical candidate mention strings that occur multiple times in the corpus may appear more than once in the ranking. We only evaluate query mentions that had a synonym present in the data, ie another (different) mention string linked to the same concept as those without synonymous mentions present cannot be matched in this dataset. We only include candidates from within the same data fold.

We scored a synonym as correct if both that mention and the query linked to the same concept in the gold annotation (Gold annotations refer to the concept provided by the expert annotators for each mention, and are the point of comparison in our work.). We measured mean reciprocal rank (MRR), coverage (percentage of time a correct synonym appeared in the ranked list), and top-1 accuracy. For the top-1 results in each model, we calculate the Jaro-Winkler distance,^28^ measuring lexical similarity, between the mention text and the synonym. The mean Jaro-Winkler distance between a mention and all of its gold label synonyms was 0.504 for the tuning and 0.476 for the test sets. Both the tuning and test sets were not used for training representations, but only the test set was held out until the end of experimentation.

### Results

Table 1 shows the results for candidate models on the tuning data. We selected the model that produced the highest MRR for each model type (noted by separators in the table), and evaluated it on the test set (Table 2). In all cases, ELMo models outperform the Word2vec, character ngram, and Context2vec, and BERT models. For MRR, the ELMo models outperform others by 0.09 in tuning and 0.12 in testing. For Top-1, ELMo provides an 11.3% increase in tuning and 13.9% increase in testing. For coverage, ELMo provides smaller but noticeable improvements—a 4.3% increase for tuning and a 4.1% increase for testing. Adding Med2vec information to the ELMo model does not provide consistent improvements to any metric, with the exception of coverage. The ELMo model with Med2vec integration provides a small increase in coverage over the standard ELMo model and the other model types. The Jaro-Winkler distance of the ELMo model varies by layer level—the lowest layer has the most lexically similar synonyms, while the higher layers have the least lexically similar synonyms of any model. Overall, we see clear benefits by moving from the type level embeddings to contextualized representations, with some benefit to incorporating patient context.

### Synonym analysis

In addition to the quantitative results, we perform a qualitative analysis of one model, ELMo (L0, Max). For 400 Top-1 errors (the mention was not matched with a synonym as the first result, but may be matched lower in the list), we categorized the error as 1 of 5 types. Correct and incorrect examples with error types are listed in Tables 3 and 4, respectively.

**Table 2.** Mean reciprocal rank, coverage, and top-1 accuracy, and Jaro-Winkler average for correct synonyms in the top-1 for pairwise identification of synonyms of the top 50 results run on the test data (*n* = 599)

Model	MRR	Coverage (%)	Top-1 Acc. (%)	JW Top-1
Word2vec	0.355	69.40	29.20	0.798
Char. Trigram	0.359	67.90	28.00	0.826
C2v + M2v	0.335	60.60	28.60	0.719
ELMo (L0, Max)	0.474	62.40	43.10	0.838
ELMo+M2v In(L0, Avg)	0.476	**73.50**	40.70	0.813
ELMo+M2v Out(L0, Max)	**0.487**	63.40	**44.70**	0.814
BERT (L1, Avg)	0.442	64.94	39.07	0.835

*Note*: Significance tests were performed using a two-sided *Z*-score test to compare the best performing models (bolded) to the baseline models. For Top-1, the best performing models is found to be significant compared to the best performing non-ELMo model. For Coverage, the increase between Word2vec and the best model is not significant. Significance testing is not performed for MRR or Jaro Winkler Top-1.

**Table 4.** Incorrect Top-1 examples are listed by category, along with the percentage of occurrence (from 400 reviewed Top-1 errors) from the ELMo (L0, Max) model tuning set results

Category	Percentage	Mention	Top-1 synonym
Synonym overlap	52%	Left atrium…dilated	Right atrium…dilated
		Myocardial infarction	Inferior myocardial infarction
		Diabetes mellitus	Diabetes mellitus type 2
		Aortic valve disease	Valvular heart disease
		Dilated RA	Dilated RV
Abbreviation	19%	LVH	MVP
		NIDDM	DM
Morph. or lexical overlap	16%	Hypokinesis	Akinesis
		Bradycardic	Tachycardic
		Cyanosis	Stenosis
No relation	9%	Nausea	Masses
		Clubbing	Bleeding
Similar concepts	5%	Bleed	Bleeding
		Cerebrovascular accident	Cerebrovascular accidents

The first and most common error type was word overlap—the mention and the incorrect synonym shared at least one word, but the remaining non-shared words contrasted the meaning of the mention and synonym. This may be due to the simple method we use to combine words into a single representation (in this case, the dimensional maximum operation). For example, for the mention “diabetes mellitus” the top synonym is “diabetes mellitus type 2”—the 2 share words and may be linked to related concepts, but the model does not put enough weight on the distinction provided by the words “type 2”.

Second, mentions with abbreviations were commonly mismatched with other abbreviations. Some abbreviations are linked to concepts that are related (eg “AR” is an abbreviation for *Aortic Valve Insufficiency* and “MR” is an abbreviation for *Mitral Valve Insufficiency*), while others share no relation (eg “UTI” is an abbreviation for *Urinary Tract Infection* and “ptx” is an abbreviation for *Pneumothorax*).

A third class of error was morphological or partial lexical overlap—the mention and incorrect synonyms do not share a word, but often shared a prefix or suffix (eg “hypokinesis” and “akinesis” share the suffix *kinesis*). Fourth, some errors consisted of mention and incorrect synonym pairs that were not correct due to annotation decisions in the data—they often have the same lexical form but are different concepts in the ontology referenced in the annotations, or may have been assigned a non-concept annotation. Finally, we could not explain some errors as there was no clear relation between the mention and the incorrect synonym.

**Figure 4.**
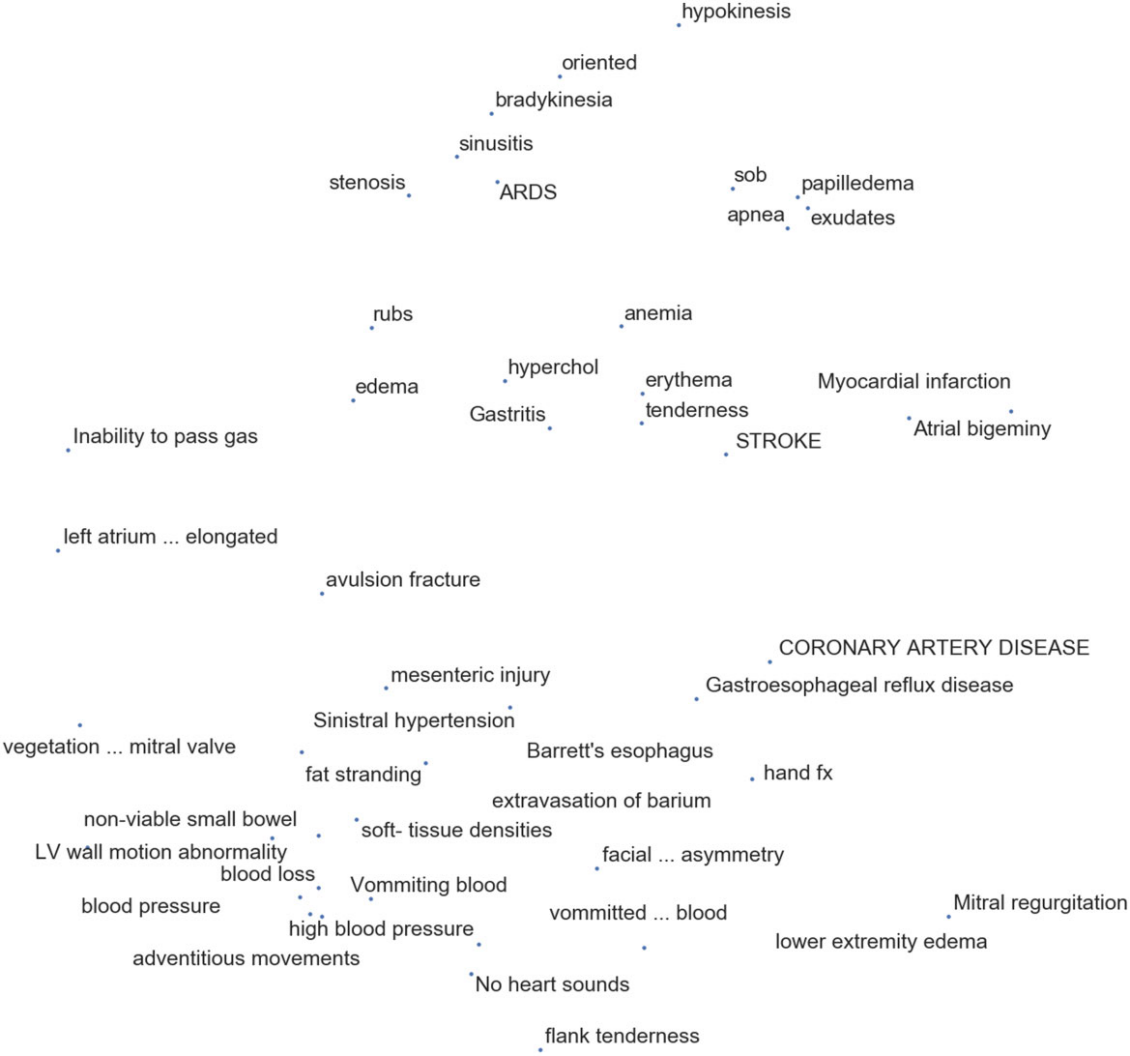
We performed dimensionality reduction using t-SNE^29^ on the tuning set mention representations from the ELMo (L0, Max) Model, randomly selected 5% of unique mention strings.

We visualize selected synonyms from the tuning set in Figure 4 using t-SNE.^29^

## SIGNIFICANCE

Models using ELMo consistently provide the best performance for using unsupervised representations for the pairwise mention synonym identification task—in MRR, Coverage, and Top-1. We attribute this to 2 factors. First, the ELMo model allows the sentence surrounding the mention to influence the final representation, which better incorporates the context in which the mention occurs. Mentions of concepts that do not share a similar lexical form may appear in similar contexts with similar words, and including the sentence allows for this to be reflected in the final representation. Second, using a character model may better handle out of vocabulary words and morphology. Integration of Med2vec into ELMo provides an improvement in coverage, which indicates that integrating patient information can better inform representation learning for this task. To explore this further, we trained an ELMo model that used tokens instead of characters, and an ELMo model that didn’t use the full sentence to build representations. In both cases, the performance was worse than the standard ELMo models, and further it wasn’t clear which is the more important factor.

While recent work has shown that BERT performs well on a variety of clinical tasks,^26^ we find that it performs slightly worse than ELMo for this task. Other work has shown that fine tuning is vital for BERT performance.^30^ This is one potential factor in the lower performance of this model. Since we assume an unsupervised setting, we cannot conduct task specific fine-tuning.

## BACKGROUND

Previous research has studied identifying medical synonyms from within the UMLS ontology using unsupervised representations, such as Wang et al^15^ using a method centered on Word2vec’s CBOW method. They augment this representation by adding a variety of rule based features, and then train a linear classifier to detect synonymy. Other work^31^ uses Random Indexing and Random permutation to identify synonyms in clinical notes and journal article data on Swedish data. Unlike Wang et al, this is an unsupervised method that uses a ranking approach but is limited by its reliance on term statistics instead of character-based representations. Related work explored applying a similar method to Japanese patient blogs.^32^ Earlier work^14^ explored retrieving synonyms for biomedical text in UMLS and other ontologies using a pattern generation algorithm. While benefiting from interpretability, this does not allow for the integration of character or contextual models that our work provides. Additional work has studied approaches to synonym expansion in non-medical domains,^10^^,^^33^ and the related tasks of addressed abbreviation and acronym resolution^34^^,^^35^ in the clinical space. There has been a wide variety of research into the related task of medical concept linking—well-known systems include cTAKES^36^ and MetaMap.^37^ Recent work includes a Sieve-Based method,^38^ and Rajani et al,^39^ which uses a stacking approach. Others have focused on concept linking for bio-medical literature^40–42^ and non-medical text.^43^^,^^44^

## CONCLUSION

Learning unsupervised representations of words in context provides an effective way to identify mention pairs that are synonyms, allowing us to leverage a large corpus of unsupervised data, and to integrate patient information from the clinical record. We expect that when integrated into a relevant downstream task, such as a statistical concept linking system, we would observe gains in the task’s performance.

## FUNDING

This work was supported by the Center for Disease to Health (CD2H) (U24 TR002306).

## AUTHOR CONTRIBUTIONS

E.S.: designed the study, wrote the manuscript, designed and implemented all methods. M.D.: designed the study, and edited the manuscript. Both authors approved the final manuscript.

## CONFLICT OF INTEREST STATEMENT

None declared.
